# How to Improve Postgenomic Knowledge Discovery Using Imputation

**DOI:** 10.1155/2009/717136

**Published:** 2009-01-11

**Authors:** Muhammad Shoaib B Sehgal, Iqbal Gondal, Laurence S Dooley, Ross Coppel

**Affiliations:** 1 ARC Centre of Excellence in Bioinformatics, Institute for Molecular Bioscience (IMB), University of Queensland, St Lucia, QLD 4067, Australia; 2 Victorian Bioinformatics Consortium, Monash University, VIC 3800, Australia; 3 Gippsland School of Information Technology (GSIT), Faculty of Information Technology, Monash University, Churchill, VIC 3842, Australia; 4 Faculty of Mathematics, Computing and Technology, The Open University, Milton Keynes MK7 6BJ, UK; 5 Department of Microbiology, Monash University, VIC 3800, Australia

## Abstract

While microarrays make it feasible to rapidly investigate many complex biological problems, their multistep fabrication has the proclivity for error at every stage. The standard tactic has been to either ignore or regard erroneous gene readings as *missing values*, though this assumption can exert a major influence upon postgenomic knowledge discovery methods like gene selection and *gene regulatory network* (GRN) reconstruction. This has been the catalyst for a raft of new flexible imputation algorithms including *local least square impute* and the recent *heuristic collateral missing value imputation*, which exploit the biological transactional behaviour of functionally correlated genes to afford accurate missing value estimation. This paper examines the influence of missing value imputation techniques upon postgenomic knowledge inference methods with results for various algorithms consistently corroborating that instead of ignoring missing values, recycling microarray data by flexible and robust imputation can provide substantial performance benefits for subsequent downstream procedures.

## 1. Introduction

The study of genes and their transactional relationship with other genes can be modelled using machine learning algorithms in a diverse range of applications from disease analysis [1] and drug progression for target diseases [2] through evolutionary study [3] and comparative genomics [4], all of which are characterised by using microarray gene expression data. The statistical analysis of microarray datasets depends highly upon the accuracy of the gene expression methods. Microarray production is a complex process, whereby samples are prepared for differential expression in a series of stages involving the laying of specimens on the slides by a robotic arm, imaging of the slides, and finally determining the numerical gene expression values. Each step inevitably exhibits a propensity for error [5], a corollary to this is the inherent erroneous gene expression values for certain genes, which are popularly referred to as *missing values*. While microarray technology is continually being refined, there is an enormous amount of public domain gene expression data available that frequently contains at least 5% erroneous spots. Indeed, in many datasets, at least 60% of genes have either one or more missing values [6], which can seriously impact on subsequent data analysis involving, for example, significant gene selection, *gene regulatory network* (GRN) reconstruction, and clustering algorithms [7, 8].

The simplest ways to address this problem are to either repeat the experiment, though this is often not feasible for economic reasons, or ignore those samples containing missing values, but again this is not recommended because of the limited number of available samples. Alternative strategies include row average/median imputation (substitution by the corresponding row average/median value) and the ubiquitous *ZeroImpute,* where missing values are replaced by zero. Both approaches are high variance, with neither exploiting the underlying data correlations which can lead to higher estimation errors [9]. The prevailing wisdom is to accurately estimate missing values by exploiting the latent correlation structure of the microarray data [8, 10], as manifested by the development of numerous microarray imputation techniques including *collateral missing value estimation* (CMVE) [11], *singular value decomposition impute* (SVDImpute) [9], *K-nearest neighbour* (KNN) [9], *least square impute* (LSImpute) [10], *local LSimpute* (LLSImpute) [8], *Bayesian principal component analysis* (BPCA) [12], a set of theoretic framework based on *projection onto convex sets imputation* (POCS Impute) method [13] and most recently, *heuristic collateral missing value imputation* (HCMVI) [14]. In addition, other methods which use contextual information include *gene ontology-based imputation* (GOImpute) [15] and metadata-based imputation technique [16].

This paper will investigate the gene expression correlation assumption by empirically analysing different postgenomic knowledge discovery methods including gene selection and GRN reconstruction techniques in the presence of missing values, specifically for the breast and ovarian cancer datasets of Hedenfalk et al. [17] and Jazaeri et al. [18], respectively. The rationale for choosing these two datasets is that generally cancerous data [19] lacks molecular homogeneity in tumour tissues, which makes missing value estimation far more challenging. Additionally, breast cancer is the second leading cause of cancer death in women today (following lung cancer), with 1 in 11 Australian women being diagnosed with the disease before the age of 75, and the number of breast cancer patients increasing everyday, as diagnosis methods improve [20]. Ovarian cancer is the fourth most common cause of cancer-related deaths in American women of all ages, as well as being the most prevalent cause of death from gynaecologic malignancies in the United States [21].

**Figure 1:**
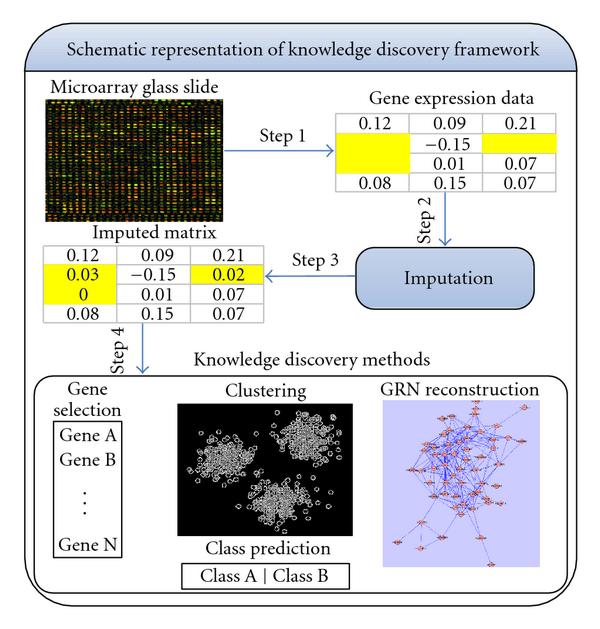
**A Schematic representation of postgenomic knowledge discovery framework**.

Figure 1 displays a generic postgenomic knowledge inference framework, with the DNA sample being firstly converted to expression values prior to any knowledge inference being undertaken. As highlighted earlier, this phase (STEP 1 in Figure 1) can introduce several erroneous (missing) values that can significantly impact upon any subsequent analysis. Unfortunately, while there have been many propitious imputation algorithmic contributions (STEP 2), there is still the pervading fallacy that either new data analysis methods will successfully manage missing values or more seriously that missing values in fact do not impact appreciably upon downstream analysis [22]. Interestingly, even though there have been some attempts to test the impact of imputation on clustering methods [23, 24], no comprehensive single study has been undertaken to date to analyse the impact missing values can have on different postgenomic knowledge discovery methods like gene selection, class prediction, clustering of functionally related genes, and GRN reconstruction (STEP 4). This paper cogently argues that imputation is both an integral and indeed mandatory preprocessing step (STEP 2) prior to applying any knowledge discovery method (STEP 4). This judgement is justified by analysing various results which consistently reveal improved estimation accuracy when missing values are approximated by more flexible approaches such as HCMVI and *LLSImpute* (STEP 3) because of their innate ability to preserve the variance of the data compared to other popular, if simpler, high-variance methods.

Aside from the obvious numerical relevance of missing value estimation, another key driver is the biological significance of imputation, particularly algorithmic performance in estimating significant genes in microarray data that may be erroneously affected. *Plakophilin 2* (PKP2), for example, is present in breast carcinoma cell lines [25] and is significant as it serves as a marker for the identification and characterisation of carcinomas derived either from or corresponding to, simple, and complex epithelia [26]. As will be witnessed in Section 6, PKP2 is often not selected by gene selection methods when missing values are present and so would generally be either ignored or replaced when conventional estimation methods are applied. By judiciously employing a flexible imputation strategy such as HCMVI, however, the probability that these genes are correctly selected can be significantly enhanced. Similarly, the GRN reconstruction performance may be significantly influenced by missing values with a substantial number of vital coregulation links being neglected when imputing by traditional and contemporary methods (Sections 3 and 4). The interaction in breast cancer data between *ADP-ribosylation* factor 3 and estrogen sulfotransferase (EST), which is similar to the NSAP1 protein, is, for instance, consistently overlooked when missing values are introduced, though they have been successfully reconstructed using flexible imputation methods (Section 5). In both scenarios, accurate imputation crucially eliminates the need for repeating an experiment which can be costly, and may be pragmatically infeasible.

This paper presents a treatise on existing imputation methods by examining their performance in managing microarray dataset missing values to improve postgenomic knowledge discovery. Concomitant with analysing the numerical accuracy of imputation, the biological significance for two proteins is analysed, namely, *KIAA1025* and *MHC*, from the breast and ovarian cancer datasets, respectively, because of their acknowledged importance in diagnosing the different cancer types [27–29].

The remainder of the paper is organised as follows. After formally defining the nomenclature, Sections 3, 4, and 5 will, respectively, review the gamut of traditional, contemporary, and flexible microarray missing value imputation algorithms together with their particular epithets and limitations. A reflective analysis is then presented in Section 6 upon a series of experiments performed on various breast and ovarian cancer microarray datasets, including both statistical and biological significance interpretations, while some conclusions are provided in Section 7.

## 2. Nomenclature

The convention adopted in all the imputation strategies is to assume that the gene expression matrix *Y* has *m* rows and *n* columns, where the rows and columns represent genes and samples, respectively, as in (1). A missing value in gene expression data *Y* for gene *i* and sample *j* is formally expressed as *Y _ij_*(1)

Imputation strategies have been broadly classified into three categories: traditional, contemporary, and flexible techniques. Original imputation approaches, which replace a missing value by either zero or row/column mean, are designated as *traditional,* as they are simple and computationally efficient, but do not take advantage of any latent correlation within the data. *Contemporary* techniques subsequently evolved to improve the estimation accuracy by using inherent data correlations, usually under the assumption that the causal correlation structure is either localised or globalised. They are also characterised by using a fixed number of predictor genes in the estimation which limits the flexibility to fully exploit any data correlations. This was the incentive for the most recent family of *flexible* imputation methods which are able to freely adapt to the data distribution by automatically determining the optimal number of predictor genes, thereby minimising the impact of missing values on subsequent biological analysis. In the following sections, these three imputation categories are, respectively, reviewed.

## 3. Traditional Imputation Techniques for Microarray Data

These are broadly characterised by replacing expression values of those genes that posses missing values by zero, their gene/sample mean or median, and in certain cases, by using the well-known KNN method. The advantages and disadvantages of these popular approaches are now discussed.

### 3.1. Zeroimpute and Mean/median Imputation

In these methods, missing values are, respectively, replaced either by zero (*ZeroImpute)* or by the gene/sample average [30] and/or median. The attraction is their simplicity and computational efficiency, though none take advantage of the underlying correlation structure of the data, with the consequence that the data variance is generally high. This means that when there are a large number of missing values present in the microarray data, these imputation strategies can significantly compromise subsequent postgenomic analysis. The impact, however, can be reduced by adapting the estimation parameters to the underlying correlation structure of the data, with the following sections examining some well-established methods.

### 3.2. Singular Value Decomposition-Based Imputation (svdimpute)

This uses the combination of *singular value decomposition* (SVD) [9] and *expectation maximization* (EM) [31] to estimate the missing values by calculating mutually orthogonal expression patterns often referred to as *Eigen genes*. As SVD calculations require the entire matrix, missing values are replaced by their row mean prior to the *k* most effective *Eigen genes* being selected according to their corresponding Eigen values. The imputed missing value estimate for *Y _ij_* is then calculated by regressing *g _i_* against the *k* most effective *Eigen genes* with expression values from sample *j* which contained the missing value being ignored. *SVDImpute* reduces imputation errors by recursively estimating the missing values using the EM algorithm until the change in the matrices becomes less than an empirically determined threshold, nominally 0.01 [9]. The technique performs best when 20% of the *Eigen genes* are used for estimation, and while it is a better strategy than high-variance approaches like *ZeroImpute*, it has the drawbacks of both being highly sensitive to noise and only considering global data correlations, which inevitably leads to higher estimation errors in locally correlated datasets.

### 3.3. K-Nearest Neighbour (knn) Estimation

KNN [9] estimates missing values by searching for the *k* nearest genes normally by applying the Euclidean distance and then taking the weighted average of these *k* genes. The *k* genes whose expression vectors are most similar to genetic expression values in all samples, except the sample which contains the missing value, are selected. The similarity measure between gene *g _i_* and other genes is then determined by the Euclidian distance over the observed components in sample *j*, and the missing value estimated as the weighted average of the corresponding entries in the selected *k* expression vectors, where the contribution of every gene is scaled by the similarity of its expression to *g _i_*.

While KNN is flexible in terms of the choice of similarity measure, it does imply the performance of a specific metric is data dependent. Troyanskaya et al. [9] demonstrated that Euclidean distance performs better than other similarity measures for microarray data, and though it is highly sensitive to microarray data outliers, log-transforming the data can significantly reduce their effect in determining gene similarity.

The choice of an appropriate *k* value especially influences imputation performance. Experimental results have established that for small datasets  is the best choice [7], while Troyanskaya et al. [9] observed that KNN is insensitive to values of *k* in the range 10 to 20. The key point to emphasise is that regardless of the underlying structure of the microarray data, a preset value of *k* is employed which clearly does not fully harness the capability of an imputation method. A much more creative strategy is to endeavour to automatically determine the best *k* value from the data correlation structure, which is the fundamental premise of the two flexible imputation techniques described in Section 5.

Summarising, while traditional algorithms have been widely adopted, the inherently high data variance has a major impact on downstream analysis methods like significant gene selection and class prediction GRN reconstruction. To relax this restriction, more robust techniques have evolved in an attempt to garner superior performance in terms of estimation accuracy, although as will be witnessed, they still exhibit some limitations, most notably from a biological significance perspective. Section 4 focuses on some of the most well-established contemporary imputation approaches.

## 4. Contemporary Imputation Techniques for Microarray Data

This category embraces those methods that implicitly attempt to lower the data variance of missing value estimates, by seeking to exploit the underlying localised or global correlation structure of the microarray data. Some of the most popular algorithms together with their relative merits and demerits will now be investigated.

### 4.1. Least Square Impute (lsimpute) Estimation

This is a regression-based method that exploits the correlation between genes. There are three variants of the imputation *LSImpute* [10] algorithm, namely, *LSImpute-Gene*, *LSImpute-Array,* and *LSImpute-Adaptive*. *LSImpute-Gene* estimates missing values using the correlation between the genes (intrasample) while *LSImpute-Array* exploits intersample correlation while *LSImpute-Adaptive* combines both techniques using a *bootstrapping* approach [32]. The communal features of all three *LSImpute* variants will now be delineated.

To estimate missing value *Y _ij_* in (1), the *k*  most-correlated genes are firstly selected, whose expression vectors are similar to gene *i* from *Y* in all samples except *j*, where all the correlated genes do not contain any missing values. As *LSImpute-Gene* is based upon a regression, it mandates that the number of model parameters must be lower than the number of observations, though in general for microarray data, the number of genes is usually much greater than the sample number. The algorithm then computes regressive estimates for each selected gene and the missing value estimate is obtained from their weighted average.

While *LSImpute-Gene* affords greater accuracy than traditional imputation methods like KNN and SVDImpute (Section 3), it still has the same fundamental limitation of using a preset *k* value. Bø et al. [10], for example, empirically determined  as the most suitable value for their particular dataset, though crucially this finding is data dependent and not generic. It also demonstrated that this imputation approach works better if missing values have been initially approximated by *LSImpute-Gene* and then refined with *LSImpute-Array*. This lowers the imputation error, though commensurately it increases the computational overhead, and since it still employs *LSImpute-Gene* prior to any estimation, the value of *k* is always fixed.

*LSImpute-Adaptive* combines the strengths of both *LSImpute-Gene* and *LSImpute-Array* by fusing their respective imputation results. It modifies the weights for each imputation using a *bootstrapping* process [32], with empirical results [10] endorsing that this strategy performs better when either variant is separately applied.

With the flexibility to adjust the number of predictor genes in the regression, *LSImpute* performs best when data exhibits a strong local correlation structure, though the comparative prediction accuracy is still inferior to that achieved by the new flexible imputation algorithms, which dynamically determine *k* directly from the data (Section 5).

### 4.2. Bayesian Principal Component Analysis (bpca) Estimation [12]

BPCA estimates missing values using Bayesian estimation theory with a variational algorithm [33] to calculate the model parameters and ultimately the imputed value *Y _ij_*. The posteriori distribution  of the missing value and the posteriori distribution  of the model parameter  are firstly computed from gene values having no missing values and since this distribution calculation requires the complete matrix, so missing values are replaced by their corresponding gene averages. The model parameters  are then used to compute the current posteriori distribution, with the *maximum likelihood* [32] parameters being iteratively updated using the current posteriori distribution of model parameters and missing values, until convergence is reached.

By considering only global correlations within a dataset, BPCA has a distinct advantage in terms of prediction speed compared with all the other imputation techniques analysed, but its performance is highly dependent on either a strong underlying global correlation within the data or having a very high number of samples. This is an offset by the likelihood of high imputation errors when either the dataset is locally correlated or comprises a small number of samples.

### 4.3. Collateral Missing Value Estimation (CMVE) [11]

This algorithm is unique in contemporary missing value imputation techniques in using multiple estimates. Like *LSImpute*, it firstly estimates the missing value *Y _ij_* by identifying the *k* most-correlated genes, with either a covariance or Pearson correlation matrix being employed, depending upon the data distribution to find these correlated genes. LS regression and two variants of the *nonnegative LS* (NNLS) algorithm are then applied to compute three separate estimates for *Y _ij_*, which are then linearly fused as follows:(2)

where , , and  are the weights assigned to each constituent imputation estimate.

CMVE uses LS regression of *k*-correlated genes for the first missing value estimate , while NNLS and linear programming compute the other two estimates  and . The rationale for including NNLS is that unnormalised microarray data has only positive values so NNLS takes advantage of exploiting the positive search space. If the data is either normalized or log-transformed then it will contain some negative values so LS regression is used for this particular estimation. Since both the Pearson correlation and the covariance functions necessitate complete imputation matrices, so CMVE firstly replaces all missing values by gene averages. Once the initial missing value estimate is generated, then new estimated value is used in all future predictions, which is a distinctive feature of this particular imputation strategy.

CMVE has been proven to perform best for locally correlated data, providing consistently superior imputation quality compared to all the aforementioned techniques, by virtue of the property of recycling estimated values in future predictions [34]. It is also more robust as witnessed by its performance in the presence of high numbers of missing values. The main drawback of CMVE, just like all the other contemporary algorithms, is the preset value of *k* which means that it does not fully adapt to the correlation structure of the data and compromises performance when data has a global structure.

In summarising the imputation methods reviewed so far, the main assumption relates to the underlying correlation structure of the dataset, where KNN, *LSImpute,* and CMVE perform better when data is locally correlated, while *SVDImpute* and BPCA are more apposite for missing value estimation in globally correlated datasets. From a postgenomic knowledge inference viewpoint, however, any estimation strategy must be adapted to the correlation data structure so imputation performs equally well for both types of correlated data. The next section presents two recent flexible imputation methods that exhibit this propitious property, in automatically adapting to the data correlation structure to produce minimal imputation error.

## 5. Flexible Imputation Techniques for Microarray Data

Flexible imputation techniques use, to some extent, core building blocks developed for their contemporary estimation counterparts in Section 4, and are characterised by automatically selecting, a priori, the optimal number of estimator genes from the data correlation structure. This avoids the problem that if the data is globally correlated, then a small number of predictor genes (low *k* value) may ignore genes that are strongly correlated to the gene having the missing value. Conversely, when an unnecessarily large value of number of genes (high *k* value) is used, this can introduce genes for prediction which either has little or no correlation to the gene with missing values. Two techniques are reviewed in this category.

### 5.1. Local Least Square Impute (llsimpute) [8]

This is similar to *LSImpute* in that it estimates missing values by constructing a linear combination of correlated genes using LS principles. The crucial difference is that in estimating *Y _ij_*, the number of predictor genes *k* is heuristically determined directly from the dataset. To determine the optimum *k*, *LLSImpute* artificially removes a known value from the most correlated gene *g _i_* before iteratively estimating it over a range of *k* values, with the *k* that produces the minimum estimation error then being used for imputation.

Kim et al. [8] employed the *L _2_* norm as well as Pearson correlation to identify the most correlated genes, with the *L _2_* norm reported to perform slightly better than the Pearson correlation method for the chosen experimental data, although the difference in prediction accuracies between the two approaches was statistically insignificant.

In comparison with the various traditional and contemporary approaches, *LLSImpute* adapts to the underlying correlated data structure, with the corollary being superior imputation performance, and while it incurs a considerably higher computational cost, from a microarray data perspective, missing value estimation accuracy always has a greater priority than computational complexity.

### 5.2. Heuristic Collateral Missing Value Imputation (hcmvi) [14]

This uses the multiestimate CMVE algorithm [11] detailed in Section 4, as its kernel building block to formulate the final imputation of missing value *Y _ij_*. It is analogous to *LLSImpute* in that it also automatically determines the optimal number of predictor genes *k* by using Monte Carlo (MC) simulation [35]. It selects multiple matrices with known gene expression values with each matrix [36] having a selection probability  in the MC simulation. HCMVI then identifies the most-correlated matrix from the Pearson correlation [37] between each selected matrix and the gene expression *Y*. These known values are then estimated by CMVE for a range of *k* values, with the optimal *k* being the one that generates the minimum estimation error.

HCMVI retains all the enhanced imputation performance characteristics and advantages of the original CMVE algorithm, while crucially automatically adapting to the underlying correlation structure of the microarray data, though as with *LLSImpute*, it incurs an additional computational overhead.

## 6. Discussion of Results

This section will rigorously examine the influence the aforementioned imputation strategies have in improving missing-value estimation accuracy for postgenomic knowledge discovery methods such as significant gene selection [38], allied with the biological significance of the imputation. Six different microarray datasets for breast and ovarian cancer tissues are used, with data being log-transformed and normalized, so that  and , in order to remove all experimental variations.

The breast cancer dataset [17] contained 7, 7, 8 samples of BRCA1, BRCA2, and sporadic mutations (neither BRCA1 nor BRCA2), respectively, while the ovarian cancer dataset [18] contained 16, 16, and 18 samples, respectively, of BRCA1, BRCA2, sporadic mutations. Each breast cancer data sample contained microarray data of 3226 genes and there were 6445 genetic expressions per sample for the ovarian dataset. It is worth noting that the number of probes in both breast and ovarian cancer datasets is different. The data are generated by different labs under different experimental conditions and thus represent experimental variations.

To equitably evaluate the performance of the traditional and contemporary imputation algorithms on downstream biological analysis methods, the number of predictor genes was fixed at  in all experiments. In contrast, the two flexible imputation methods (*LLSImpute* and HMCVI) automatically determine *k* by adapting to the correlation structure of the data. Also in this empirical analysis, the *LLSImpute* variant based upon the *L _2_* norm is applied due to its superior performance [8]. In the next section, the influence of imputation on both *significant gene selection* and GRN reconstruction (STEP 4 in Figure 1) is investigated.

### 6.1. Imputation and Biological Significance of Selected Genes

To explore the impact of each estimation algorithm upon significant gene selection, a set of genes () has been chosen from the original dataset using the *between sum of squares to within sum of squares* method which identifies genes that concomitantly have large interclass and small intraclass variations. The main reason for adopting this particular method is its proven superior performance capability to select significant genes compared with other popular methods such as the *t*-test [39]. To assess the effect of missing values on gene selection, experiments were performed across a missing value range of probabilities from .01 to .2, with values being iteratively removed from the original gene expression in (1). These were then estimated using *ZeroImpute*, KNN, *LLSImpute*, BPCA, CMVE, and HCMVI, respectively, to form  prior to being applied to selected sets of *p* genes using BSS/WSS, for each respective estimation matrix. The selected genes have been then compared with  to obtain the *true positive* percentage accuracy (*%Accuracy*) metric, to provide a dispassionate measure of the estimation performance of each algorithm.

**Figure 2:**
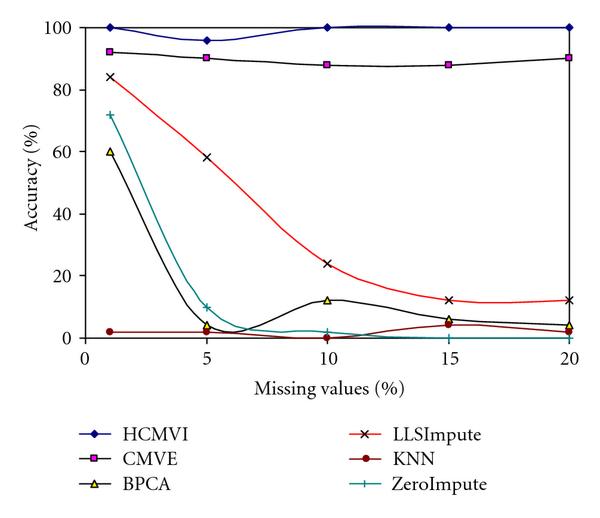
**Gene selection accuracy for 50 significant genes in breast cancer**.

**Figure 3:**
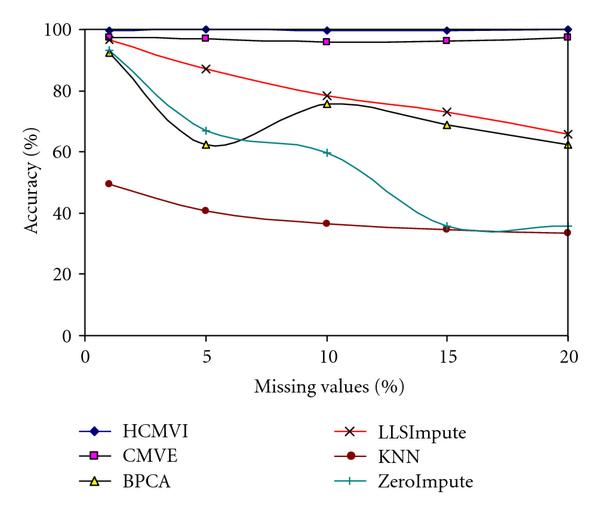
**Gene selection accuracy for 1000 significant genes in breast cancer**.

**Figure 4:**
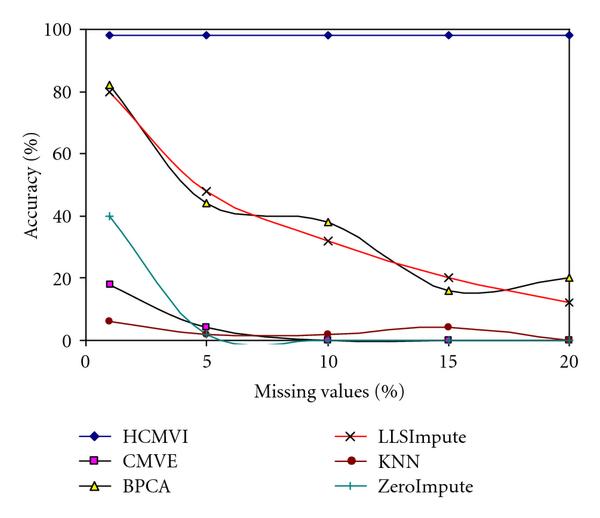
**Gene selection accuracy for 50 significant genes in ovarian cancer**.

**Figure 5:**
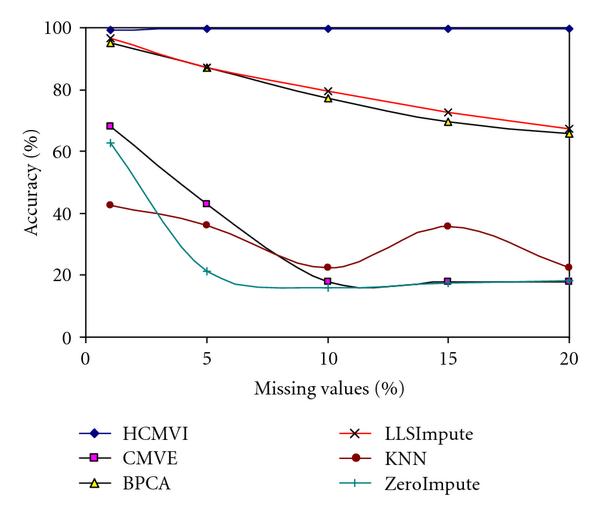
**Gene selection accuracy for 1000 significant genes in ovarian cancer**.

To eliminate performance variations with respect to the number of selected genes in the BSS/WSS method, each imputation technique was tested for 50 and 1000 significant genes, with the results in Figures 2, 3, 4, and 5 displaying the respective gene selection performance for both the breast and ovarian cancer datasets. These clearly reveal that the flexible imputation methods (*LLSImpute* and HCMVI) consistently produce superior performance for both cancer datasets, with HCMVI provides the highest *%Accuracy* metric in the experiments. In contrast, contemporary imputation algorithms like CMVE and BPCA were unable to maintain their performance across both datasets, though interestingly, CMVE performed better than *LLSImpute* as well as all the other contemporary imputation methods for the breast cancer dataset, which has a predominantly localised data correlation structure. This was not, however, maintained for the more globally correlated ovarian cancer dataset, where BPCA performed better, though it correspondingly failed to sustain the improved estimation accuracy for the breast cancer data. Not surprisingly, the high-variance traditional imputation approaches such as *ZeroImpute* and KNN exhibit the poorest performance in Figures 2–5 for both cancer datasets, confirming the judgement that incorrectly imputed missing values can have a significant potential impact upon overall gene selection performance.

Imputation algorithm performance has normally only been assessed numerically, with considerable debate within the research community of the suitability of standard evaluation measures, such as *normalised RMS error* (NRMSE). Interpreting the results from a biological significance perspective has not received the same attention, though the impact of missing values on selected genes in postgenomic knowledge discovery is clearly a major factor in algorithmic performance assessment.

### 6.2. Biological Significance of Imputation

While the primary focus is on the estimation accuracy of an imputation method, it is equally important to conduct an investigation into the biological significance of certain selected genes for the respective datasets when evaluating the impact of missing values on gene selection. Indeed, it is constructive to ascertain whether a particular imputation technique assists the gene selection methods in identifying known and novel genes for a given sample. This may provide not only valuable information for the design of basic mechanistic, diagnostic, and biomarker studies, but also valuable data for use in the construction of gene networks and pathways involved in processes like oncogenesis and resistance to tumour induction.

In examining the results for both the breast and ovarian cancer datasets, a number of genes were overlooked using traditional methods, when missing values were introduced and processed, which independent experiments [40] have confirmed alter expressions in tumor lines and so can be very important in oncogenesis. This set of genes have not only been selected by the BSS/WSS algorithm, but have been revalidated using the modified *t*-test with greedy pairs method [41] which minimizes the bias of the gene selection strategy towards either a particular imputation technique or a set of genes.

As the results for various gene selection algorithms in Table 1 reveal that the KIAA1025 protein was not always correctly selected when missing values were imputed using KNN, BPCA CMVE, and LLSImpute, but were consistently identified by HCMVI. This is a vital protein which is coregulated with estrogen receptors for both in vivo and clinical data, which are expressed in more than 66% of human breast tumors [29]. Another gene always selected by HCMVI across the range of missing values is *plakophilin 2* (PKP2) which is a common protein and exhibits a dual role, appearing as both a constitutive karyoplasmic protein and a desmosomal plaque component for all the desmosome-possessing tissues and cell culture lines. The gene is found in breast carcinoma cell lines [25] and, furthermore, because of its significance, it can serve as a marker for the identification and characterisation of carcinomas derived either from or corresponding to, simple, or complex epithelia [26].

**Table 1:** KIAA1025 and plakophilin2 selection in breast cancer dataset across the range of missing values

% MV	HCMVI	CMVE	LLSImpute	BPCA	KNN	ZeroImpute
1	KIAA1025	KIAA1025	KIAA1025			KIAA1025
	Plakophilin2	Plakophilin2				
5	KIAA1025	KIAA1025	KIAA1025			KIAA1025
	Plakophilin2	Plakophilin2				
10	KIAA	KIAA				
	Plakophilin2	Plakophilin2				
15	KIAA1025	KIAA1025				
	Plakophilin2	Plakophilin2				
20	KIAA1025					
	Plakophilin2					

Similar observations can be drawn from the study of significant genes in the ovarian cancer dataset in Table 2. For instance, MHC Class II = DQ alpha (MHC) and MHC Class II = DQ beta (MHC) genes are linked to the immune system and have been shown to be downregulated for ovary syndrome [27]. The *allele* gene is also present at a higher frequency in patients with malignant melanoma than in Caucasian controls. These genes help in particular to diagnose melanoma patients in the relatively advanced stages of the disease and/or patients who are more likely to have a recurrence [28]. The results confirm that these genes have been correctly identified by the flexible HCMVI method, while being consistently overlooked by other techniques, most notably by all traditional imputation algorithms, for missing values probabilities greater than .05.

**Table 2:** MHC class II = DQ alpha (MHC and MHC Class II = DQ beta (MHC) selection in ovarian cancer across the range of missing values

% MV	HCMVI	CMVE	LLSImpute	BPCA	KNN	ZeroImpute
1	MHC	MHC	MHC	MHC		MHC
	MHC					
5	MHC	MHC				
	MHC					
10	MHC					
	MHC					
15	MHC					
	MHC					
20	MHC					
	MHC					

Interestingly, for both cancer datasets, across the full missing value range from 1% to 20%, these regulated genes have been correctly identified when gene selection has been preceded by HCMVI imputation as confirmed in Tables 1 and 2. It highlights that consideration of the biological significance of any imputation is extremely important and underscores the need for accurate estimation prior to gene selection, particularly in the presence of higher numbers of missing values.

As alluded earlier, existing GRN reconstruction methods conventionally replace missing values by either *ZeroImpute* or gene average [30, 42], despite both inevitably impacting upon subsequent GRN reconstruction, as will now be more fully examined.

### 6.3. Impact of Missing Values on Gene Regulatory Network Reconstruction

To evaluate the influence of missing values, the *algorithm for the reconstruction of accurate cellular networks* (ARACNE) [43] has been employed because it affords better performance over alternative approaches like Bayesian networks [44] and has been tested for mammalian gene network reconstruction and compared with other techniques that are normally applied to simple eukaryotes such as *Saccharomyces cerevisiae* [45].

ARACNE firstly computes the statistical significant gene-gene coregulation using mutual information before applying a data processing inequality to prune indirect relationships, that is, genes which are coregulated by either one or more intermediate genes. To comparatively evaluate the respective imputation performances on GRN reconstruction, the number of *conserved links* is determined, which represents whether a particular coregulation link is present in both  and . The gene network  is then initially constructed using ARACNE from the original data *Y* with no missing values. As in the previous experiments, up to 20% missing values have been randomly introduced and then, respectively, estimated using traditional, contemporary, and flexible imputation methods (Section 3–5, resp.). The corresponding gene networks  are then constructed from the imputed data and  and  compared to ascertain the *conserved links*.

**Figure 6:**
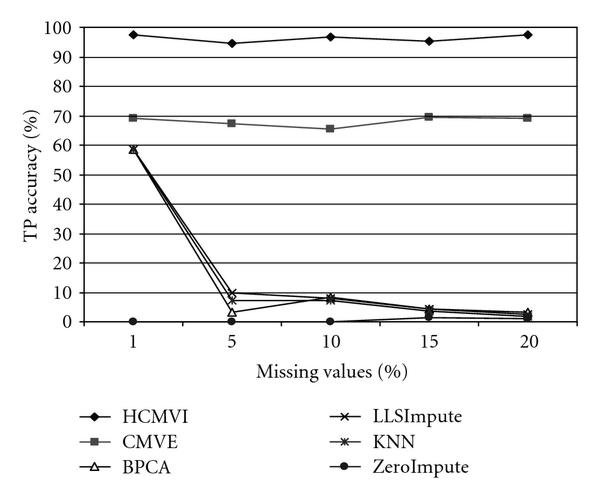
**Accuracy of conserved links in BRCA1-breast cancer data**.

**Figure 7:**
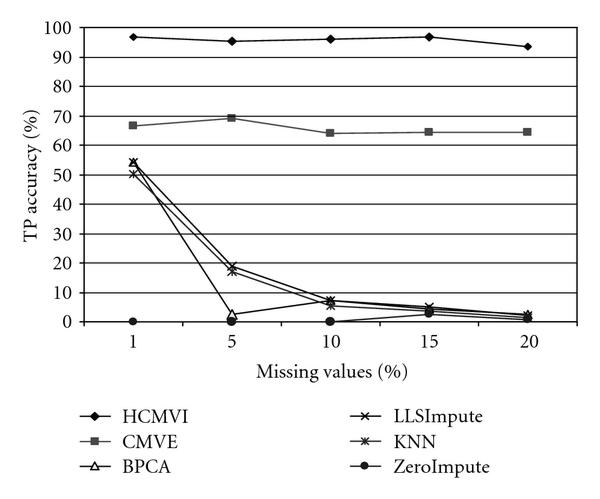
**Accuracy of conserved links in sporadic-breast cancer data**.

**Figure 8:**
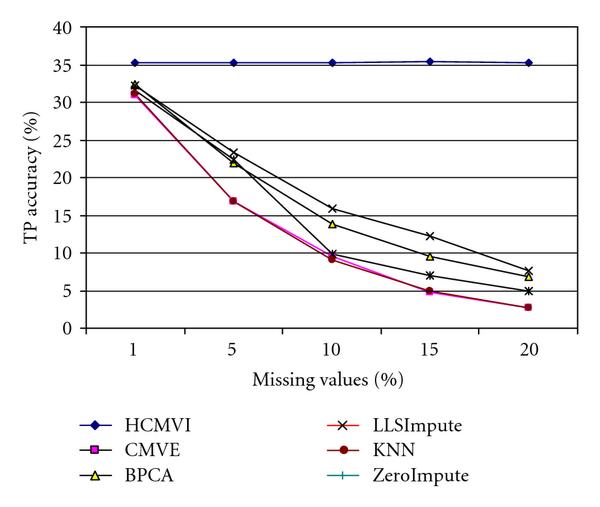
**Accuracy of conserved links in BRCA1-ovarian cancer data**.

**Figure 9:**
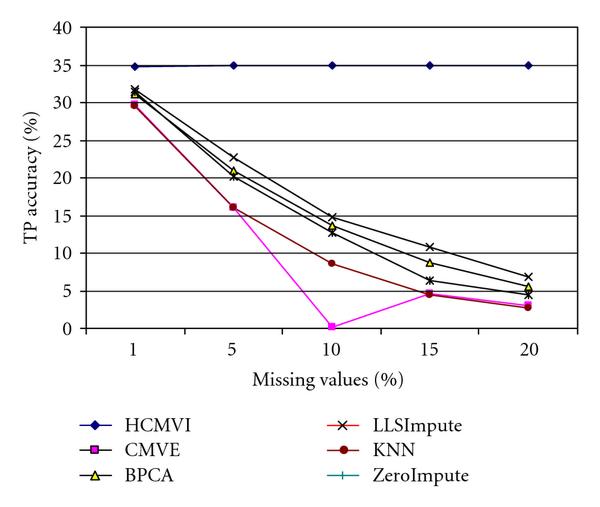
**Accuracy of conserved links in BRCA2-ovarian cancer data**.

Figures 6, 7, 8, and 9 show that the ARACNE method, which has been reported to be robust [46] for GRN construction, does not maintain its performance in the presence of missing values, especially for *ZeroImpute*. In contrast, when a flexible imputation method like HCMVI is applied, ARACNE conserves the number of links even at higher missing value probabilities. For example, in BRCA1 breast cancer data, the transcriptional link between ADP-ribosylation factor 3 (ARF3) and general transcription factor II, i, pseudogene 1(GTF2IP1) was overlooked when missing values were imputed by all traditional and contemporary methods, but was correctly inferred when values were imputed by both HCMVI and *LLSImpute*. Similarly, the link between HS1 binding protein and mitogen-activated protein kinase 3 in BRCA2 breast cancer data was reconstructed when values were imputed using HCMVI, but was neglected by all other techniques. The results for breast cancer sporadic data revealed similar observations, with, for example, the interaction between ADP-ribosylation factor 3 and EST, which is very similar to the NSAP1 protein, being identified when data was imputed using flexible methods, while being missed by the other strategies, so corroborating the importance of accurate imputation in improving GRN reconstruction performance.

In the ovarian cancer dataset, the interaction link between Ro ribonucleoprotein autoantigen (Ro/SS-A) = autoantigen calreticulin and Glutathione S-transferase theta 1 was not identified in BRCA1-data, when missing values were introduced but was regenerated when these missing values were imputed using HCMVI. Similarly, coregulation between Inhibitor of DNA binding 3, dominant negative helix-loop-helix protein, and p53 in BRCA2 ovarian cancer dataset was also missed, but the link was reconstructed when HMCVI imputation was applied across the range of missing values. In the sporadic ovarian cancer dataset, transcriptional links between CD97 and RAB-10 were again only successfully reconstructed using HCMVI, while they were overlooked by all other estimation methods again underpinning the significance of accurate missing value imputation prior to GRN reconstruction.

**Figure 10:**
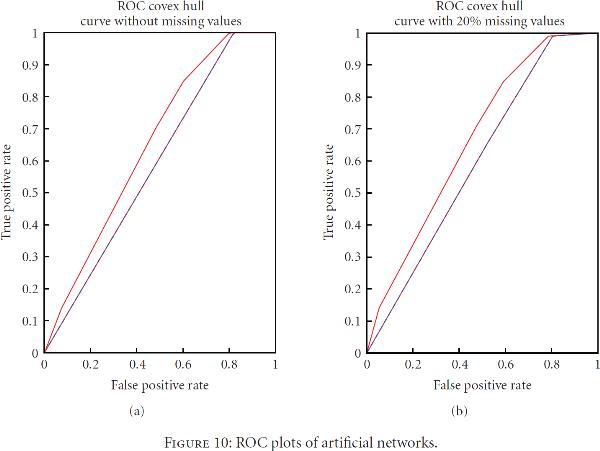
**ROC plots of artificial networks**.

The impact of missing values on GRN was further investigated on artificially created networks. Two artificial expression datasets and networks by Bansal et al. [47] were used for this purpose. Each expression data had 100 probes with 100 samples per probe. The networks were constructed using ARACNE with no imputation and compared against artificial networks to compute reference area under *receiver operating characteristic* (ROC) curve. Then, 20% missing values were introduced and imputed using HCMVI which was followed by network reconstruction using ARACNE under same experimental setup to compute area under ROC curve. Figure 10 shows average ROC curve for 10 runs with and without imputation. The areas under ROC curve for networks 1 and 2 were 0.6653 and 0.5979, respectively, when networks were constructed from complete dataset. The average areas under ROC were 0.6653 and 0.5901, respectively, when networks were constructed after randomly introducing 20% missing values and estimation using HCMVI. Again, the result shows that network inference performance is upheld if accurate imputation is used prior constructing networks.

### 6.4. Significance Test Results

**Figure 11:**
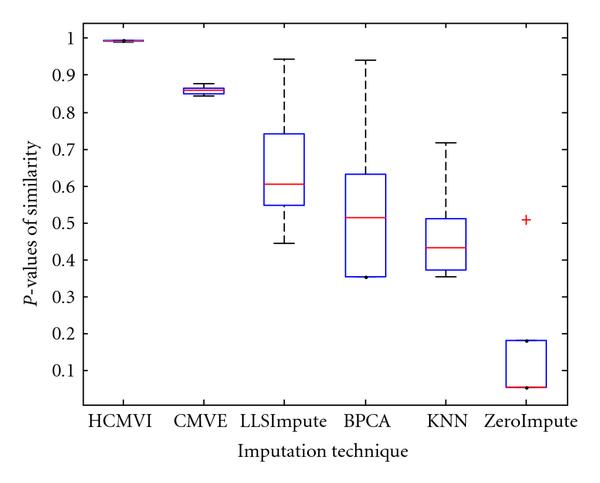
**Significance test results for BRCA1-breast cancer data**.

**Figure 12:**
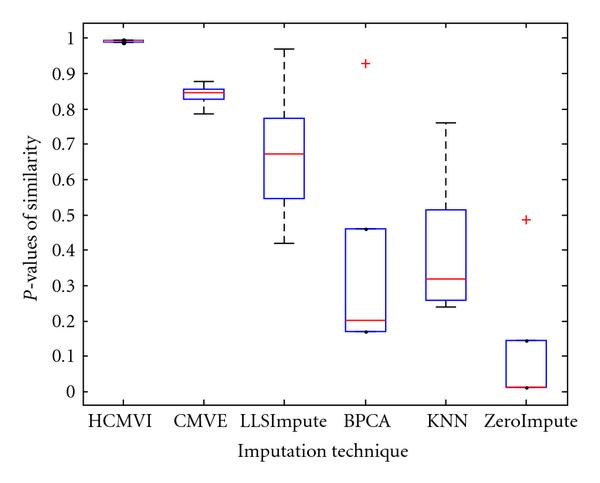
**Significance test results for BRCA2-breast cancer data**.

**Figure 13:**
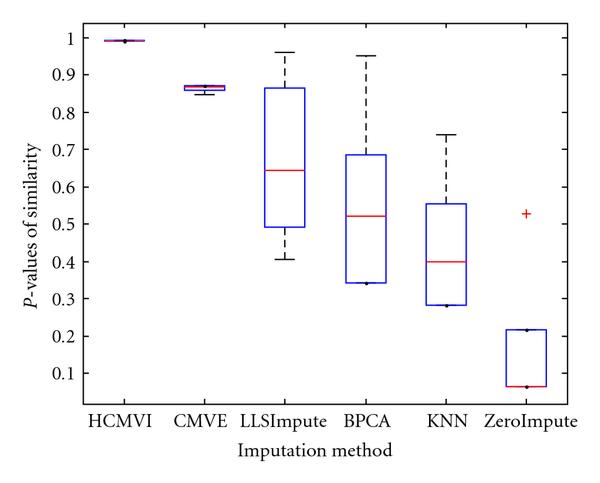
**Significance test results for sporadic-breast cancer data**.

**Figure 14:**
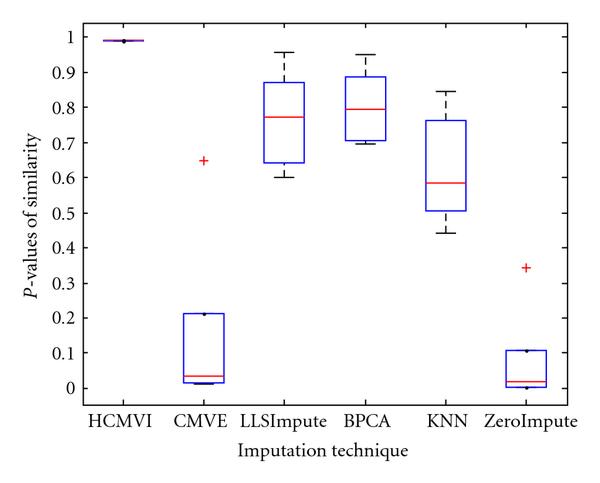
**Significance test results for BRCA1-ovarian cancer data**.

**Figure 15:**
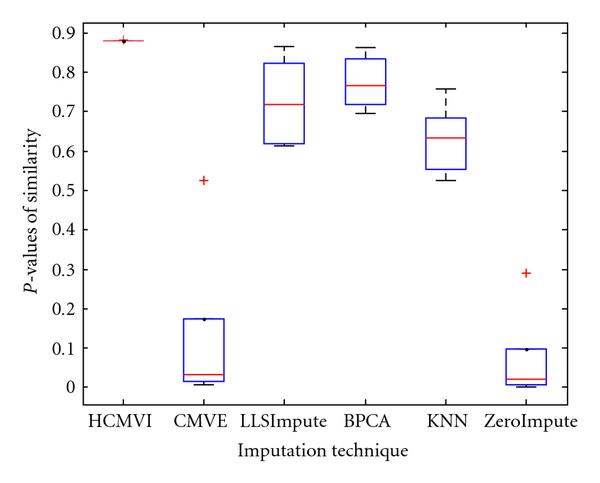
**Significance test results for BRCA2-ovarian cancer data**.

**Figure 16:**
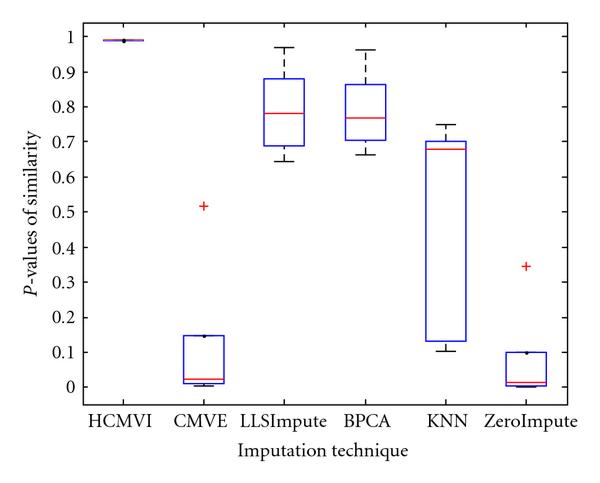
**Significance test results for sporadic-ovarian cancer data**.

For completeness, the statistical significance and variance stability of all the various imputation methods have been analysed using the *two-sided Wilcoxon rank sum statistical significance* test. The impetus for applying this test is that it does not assume that data is coming from same distribution, which is particularly important given the data variance can be appreciably disturbed by erroneous estimation, as, for instance, in *ZeroImpute*. To test the hypothesis *H _0_*, , where *Y* and  are the actual and estimated matrices, respectively, the *P*-value of the hypothesis is determined(3)

where *y _r_* is the sum of the ranks of observations for *Y* and *R* is the corresponding random variable. The corresponding results shown in box plot in Figures 11, 12, 13, 14, 15, and 16 demonstrate that traditional approaches tend to rapidly degrade at higher numbers of missing values, while both contemporary and flexible imputation techniques maintain a far more consistent performance across the range of missing values, see notably in Figures 12 and 14. As box plot can be used to display smallest observation, lower quartile, median, upper quartile, and largest observation, and it can also show if any value is an outlier. This corroborates the fundamental hypothesis that a suitably accurate imputation strategy should always be employed for microarray data before any biological downstreaming analysis is undertaken.

### 6.5. Normalized Root Mean Square Error

For completeness, the estimation performance of HCMVI and comparative imputation methods was also analysed using the traditional parametric *normalised root mean square* (NRMS) error measure, despite its limitations in reflecting the true impact of missing values on subsequent biological analysis. NRMS Error is defined as(4)

where *Y* is the original data matrix and  is the estimated matrix using HCMVI, CMVE, BPCA, LLSImpute, and KNN, respectively. This particular measure has been used by Sehgal et al. [11], Ouyang et al. [48], and Tuikkala et al. [6] for error estimation because  for zero imputation.

**Figure 17:**
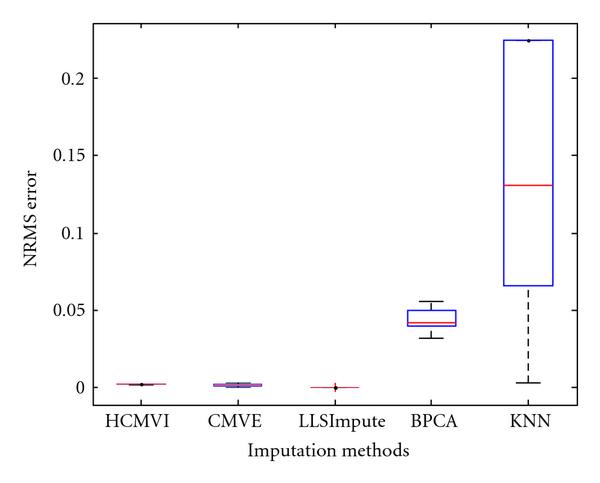
**NRMS error in BRCA1-breast cancer data**.

**Figure 18:**
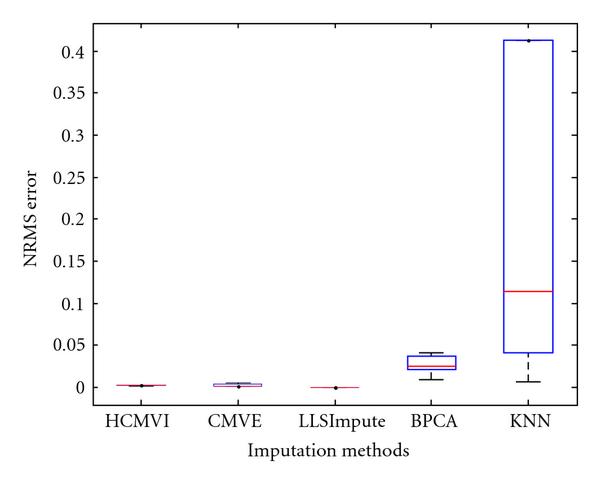
**NRMS error in BRCA2-breast cancer data**.

**Figure 19:**
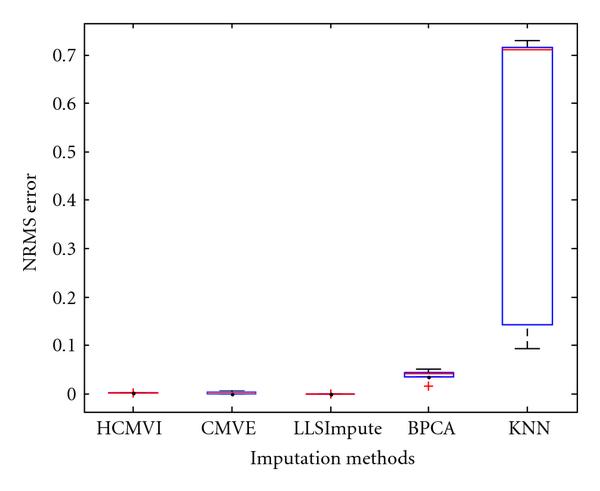
**NRMS error in sporadic-breast cancer data**.

**Figure 20:**
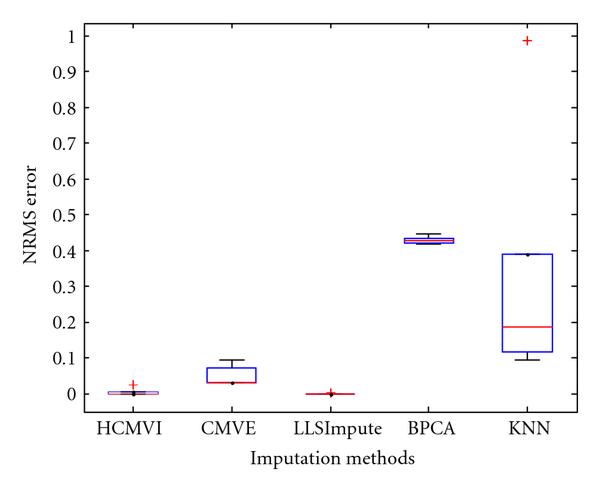
**NRMS error in BRCA1-ovarian cancer data**.

**Figure 21:**
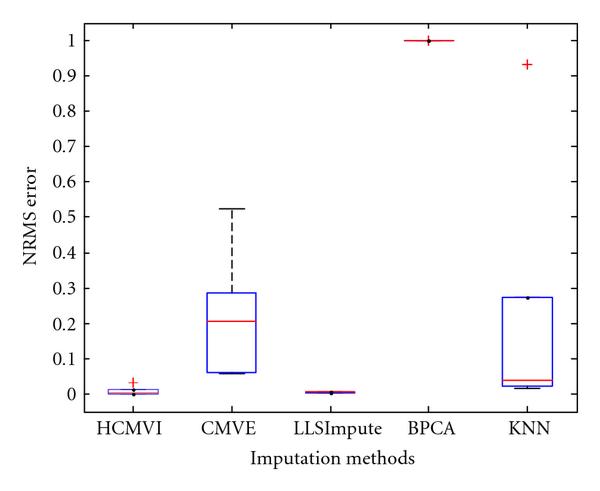
**NRMS error in BRCA2-ovarian cancer data**.

**Figure 22:**
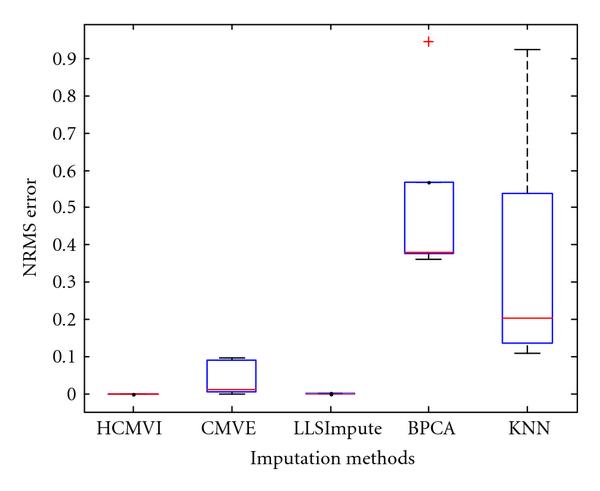
**NRMS error in sporadic-ovarian cancer data**.

Figures 17, 18, 19, 20, 21, and 22 show box plot of NMRS error for different imputation algorithms (see Supplementary Material available online at doi: 10.1155/2009/717136 for the rest of the results). It again confirms the better performance of HCMVI (see notably Figure 19) and reiterates the value of accurately exploiting information about the underlying correlation structure of the data instead of using a preset value. Interestingly, LLSImpute exhibited similar performance to HCMVI so justifying the merit of using other metrics to dispassionately compare the performance of different imputation strategies.

## 7. Conclusion

This paper has pragmatically argued that imputation can be effectively applied to recycle microarray data and in doing so provide many potential benefits ranging from cost savings to performance enhancements in postgenomic knowledge discovery. While cognisance is made that *ZeroImpute* and other traditional missing value imputation strategies are straightforward to implement, new flexible methods have been proven to exhibit much superior accuracy and performance from both a statistical and biological significance perspectives, by virtue of their innate ability to exploit any underlying data correlation structures. A comprehensive study of missing values in microarray data has been presented and their subsequent impact upon postgenomic knowledge discovery methods, including significant gene selection and *gene regulatory network* reconstruction, has been investigated. Empirical analysis has consistently shown that rather than merely ignoring missing values, which has been the preferred approach to resolve this problem, flexible and robust imputation algorithms afford considerable performance benefits and so should, wherever possible, be mandated prior to any knowledge inference process using microarray data.
